# Understanding Brain, Mind and Soul: Contributions from Neurology and Neurosurgery

**DOI:** 10.4103/0973-1229.77431

**Published:** 2011

**Authors:** Sunil K. Pandya

**Affiliations:** * *M.S. Neurosurgeon, Jaslok Hospital & Research Centre, Dr. G. V. Deshmukh Marg, Mumbai, 400026.*

**Keywords:** *Brain*, *Brainstem*, *Mind*, *Soul*, *Neurology*, *Neurosurgery*, *Philosophy*

## Abstract

Treatment of diseases of the brain by drugs or surgery necessitates an understanding of its structure and functions. The philosophical neurosurgeon soon encounters difficulties when localising the abstract concepts of mind and soul within the tangible 1300-gram organ containing 100 billion neurones. Hippocrates had focused attention on the brain as the seat of the mind. The tabula rasa postulated by Aristotle cannot be localised to a particular part of the brain with the confidence that we can localise spoken speech to Broca’s area or the movement of limbs to the contralateral motor cortex. Galen’s localisation of imagination, reasoning, judgement and memory in the cerebral ventricles collapsed once it was evident that the functional units–neurones–lay in the parenchyma of the brain. Experiences gained from accidental injuries (Phineas Gage) or temporal lobe resection (William Beecher Scoville); studies on how we see and hear and more recent data from functional magnetic resonance studies have made us aware of the extensive network of neurones in the cerebral hemispheres that subserve the functions of the mind. The soul or atman, credited with the ability to enliven the body, was located by ancient anatomists and philosophers in the lungs or heart, in the pineal gland (Descartes), and generally in the brain. When the deeper parts of the brain came within the reach of neurosurgeons, the brainstem proved exceptionally delicate and vulnerable. The concept of brain death after irreversible damage to it has made all of us aware of ‘the cocktail of brain soup and spark’ in the brainstem so necessary for life. If there be a soul in each of us, surely, it is enshrined here.

## Introduction

Millennia ago, we embarked on a quest for knowledge of the wonderful structure of man. The organ that puzzled earlier observers most was the human brain. Despite our many explorations, we remained in awe of this organ.

The evolution of our knowledge of the structure and function of the brain has been amply documented in volumes ranging from McHenry’s revision of Dr. Fielding Garrison’s work in 1969 (McHenry, 1969) to the more recent *History of Neurology*, edited by Finger and colleagues (Finger *et al*., 2009). Dr. Susan Greenfield’s book (Greenfield, 1997), intended for the lay person, embodies much useful information. We are now aware of nerve cells, their connections and their modes of communication amongst themselves and with a variety of other structures.

Injury to, and disease in, the brain often provides crucial insights on the role of its different parts. A dramatic example is the injury suffered by American railway foreman, Phineas Gage in 1848. Before his accident, Gage was liked by friends and acquaintances who considered him to be honest, trustworthy, hard working and dependable. A freak accident caused a metal tamping rod to enter under his left zygomatic arch and exit through the top of his skull (Barker, 1995).

The accident left him with little if any intellectual impairment but after the accident, Gage became vulgar, irresponsible, capricious and prone to profanity. The company that had previously regarded him as the most efficient and capable of their employees dismissed him from his job. His change in character after the accident made this the index case for personality change due to frontal lobe damage. Subsequent studies (See, for example, Blumer and Benson, 1975) have shown a wide spectrum of abnormal behaviour (compulsive and explosive actions, lack of inhibition, unwarranted maniacal suspicion and alcohol and drug abuse) after injuries to and disease in the frontal or temporal lobes and their pathways to the deeper regions of the brain.

Similar abnormalities also follow chemical derangements in the brain.

Modern marvels such as computerised tomography and magnetic resonance imaging of the nervous system have provided significant additional data. Functional magnetic resonance imaging now allows us to further localise function within the structure of the brain and correlate abnormalities of its structure and function.

Even so, two entities remain enigmatic: the mind and the soul. Where are they located? Do they lie within the brain? Since neurophysicians treat patients with a wide variety of abnormalities of the brain and neurosurgeons lay bare the brain and often work in its interior, can they provide insights?

Neurologists and neurosurgeons rank high among scientists participating in philosophical debates about what might extend beyond the physical world. They are constantly dealing with patients who have fallen into the deep hole of unconsciousness. In their attempts at restoring normalcy to bodies and minds, they also grapple with life and death. Inevitably, they ponder spirituality and the dominion of the soul.

## The Mind

*We are embodied spirits and inspirited bodies, (or, if you will, embodied minds and minded bodies). (Anonymous,* 2003)

Mind has been variously defined as that which is responsible for one’s thoughts and feelings, the seat of the faculty of reason or the aspect of intellect and consciousness experienced as combinations of thought, perception, memory, emotion, will and imagination, including all unconscious cognitive processes. The term is often used to refer, by implication, to the thought processes of reason. [See, for example, definitions of mind in a) http://en.wikipedia.org/wiki/Mind, and b) http://www.google.co.in/search?hl=enanddefl=enandq=define:mindandsa=Xandei=l973TOviFYusrAf-hfzvDwandved=0CBYQkAE]

Prioreschi (1996) concluded that by the end of the 5^th^ century B.C., the question of whether the heart or the brain was the seat of intelligence remained unresolved in Western medicine. This changed with the works of Hippocrates (ca. 460 BC–ca. 370 BC), ‘a figure of heroic proportions even if dimmed by the mist of time.’ Hippocrates’ oft-quoted statements show a clear understanding of the role of the brain vis-à-vis the mind:

*‘Men ought to know that from the brain, and from the brain alone, arise our pleasures, joys, laughter and jests, as well as our sorrows, pains, griefs and tears. Through it, in particular, we think, see, hear and distinguish the ugly from the beautiful, the bad from the good, the pleasant from the unpleasant… I hold that the brain is the most powerful organ of the human body… wherefore I assert that the brain is the interpreter of consciousness…’* (Hippocrates*: On the sacred disease*. Quoted by Prioreschi [1996])

In talking of the brain as an organ, Hippocrates very clearly refers to those functions which we ordinarily include in our understanding of the ‘mind.’ He talks of emotive mental functions like pleasures, joys, laughter and jests, sorrows, pains, griefs and tears; cognitive mental functions like thinking and seeing; aesthetic mental functions like distinguishing the ugly from the beautiful, the pleasant from the unpleasant and ethical functions like distinguishing the bad from the good–all these as attributes of the brain, and brain alone. By which he really makes a clear connection between mental functions as we understand them (‘mind’) and the structure that produces it (brain).

In his book *De anima* (*On the soul*), Aristotle (384 BC–322 BC) felt that man is born with a blank slate (*tabula rasa*) on which experiences and perceptions are written to form the mind. Although *tabula rasa* is a concept traditionally attributed to Locke, Aristotle first referred to it. See Part 4 of Aristotle’s ‘On the soul’, the second-last paragraph.(Aristotle, 2009):

‘Have not we already disposed of the difficulty about interaction involving a common element, when we said that mind is in a sense potentially whatever is thinkable, though actually it is nothing until it has thought? What it thinks must be in it just as characters may be said to be on a writing tablet on which as yet nothing actually stands written: this is exactly what happens with mind.’

Over the centuries that followed Avicenna (981–1037), Ibn Tufail (c. 1105–1185), Thomas Aquinas (ca. 1225–1274), Thomas Hobbes (1588–1679), John Locke (1632–1704), Sigmund Freud (1856–1939) and others commented on this theme. (See Trimble, 2007.)

Jean Fernel (1496–1558) treated mind and brain together in his *Physiology*. He felt that the brain refined the animal spirits. Purged of all corporeal dross, they became concepts, finally even universal concepts and the ideas of the moral values (Sherrington, 1946).

The British neurophysiologist Charles Scott Sherrington (1857–1952), ‘the scientist’s philosopher’ (Breathnach, 2004), pondered the location and functions of the mind. He acknowledged the problems encountered in attempting to restrict the mind to the brain. ‘It seems ludicrous to range such a paucity of nerve-process alongside the manifold variety of mind.’ He was well aware that ‘…our mental experience is not open to observation through any sense organ …’ He concluded that ‘The brain is the provider of mind… The mental action lies buried in the brain … in that part most deeply recessed from the outside world, that is furthest from input and output…’ (Zeman, 2007).

Pinker (2003) has recently discussed the role of nature vs nurture in the development of the mind. Dismissing the concept of the blank slate, Pinker wrote: ‘The mind cannot be a blank slate, because blank slates don’t do anything… The inscriptions (on such a slate) will sit there forever unless something notices patterns in them, combines them with patterns learned at other times, uses the combinations to scribble new thoughts onto the slate, and reads the results to guide behaviour toward goals. Locke recognized this problem and alluded to something called *the understanding*, which looked at the inscriptions on the white paper and carried out the recognizing, reflecting, and associating.’ He concluded that ‘The mind is a complex system composed of many interacting parts.’

Neurologists and neurosurgeons see patients with injured or diseased brains. Neurosurgeons attempt restoration of the internal structure of the brain to normalcy or correct disordered function in select areas by such modes as deep brain stimulation or ablation. Some operations are performed on patients who are awake. Observations on patients provided clues to the functions of the mind in relation to the structure of the brain. ‘When a surgeon sends an electrical current into the brain, the person can have a vivid, lifelike experience. When chemicals seep into the brain, they can alter the person’s perception, mood, personality, and reasoning. When a patch of brain tissue dies, a part of the mind can disappear: a neurological patient may lose the ability to name tools, recognize faces, anticipate the outcome of his behaviour, empathize with others, or keep in mind a region of space or of his own body… Every emotion and thought gives off physical signals, and the new technologies for detecting them are so accurate that they can literally read a person’s mind and tell a cognitive neuroscientist whether the person is imagining a face or a place. Neuroscientists can knock a gene out of a mouse (a gene also found in humans) and prevent the mouse from learning, or insert extra copies and make the mouse learn faster. Under the microscope, brain tissue shows a staggering complexity—a hundred billion neurons connected by a hundred trillion synapses—that is commensurate with the staggering complexity of human thought and experience… And when the brain dies, the person goes out of existence’ (Pinker, 2003).

Studies on patients who have suffered brain injury (such as Phineas Gage) have also provided interesting clues on the mind in relationship to the brain. We now know that damaged frontal lobes can no longer exert inhibitory influences on the limbic system with consequent aggressive acts.

The relation between the amount of grey matter in the frontal lobes and intelligence; the inferior parietal lobules and spatial reasoning and intuitions on numbers (as in Albert Einstein) and the third interstitial nucleus in the anterior thalamus and homosexuality (Pinker, 2003) are a few more examples of specific areas of the brain linked to characteristics attributed to the mind. Paul Broca showed that damage to the area (subsequently named after him) in the dominant cerebrum results in an inability to talk. Subsequent studies showed several other areas within the cerebrum that govern other aspects of speech.

Bilateral frontal lobotomy and subsequent more sophisticated variants such as stereotaxic amygdalotomies or cingulotomies reduce an aggressive, maniacal individual to docility (Heller *et al*., 2006).

Dr. Wilder Penfield (1891–1976), Canadian neurosurgeon, was known for his groundbreaking work on epilepsy. He operated on patients with intractable epilepsy using local anaesthesia, ensuring that they remained awake throughout the operation. He stimulated areas of the brain surface in these patients in order to demarcate the part producing epilepsy. In many patients, electrical stimulation of certain areas of the brain triggered vivid memories of past events. One patient, while on an operating table in Montreal, Canada, remembered laughing with cousins on a farm in South Africa.

Penfield concluded: ‘This is a startling discovery. It brings psychical phenomena into the field of physiology. It should have profound significance also in the field of psychology provided we can interpret the facts properly. We have to explain how it comes about that when an electrode (producing, for example, 60 electrical impulses per second) is applied steadily to the cortex it can cause a ganglionic complex to recreate a steadily unfolding phenomenon, a psychical phenomenon.

‘It is obvious that there is, beneath the electrode, a recording mechanism for memories of events. But the mechanism seems to have recorded much more than the simple event. When activated, it may reproduce the emotions which attended the original experience. What is more, the ganglionic mechanism continues to add to itself the memory of emotions which attend the recollection of the event and the substance of the man’s reasoning regarding the significance of the event…

‘The neuronal mechanism which we have stumbled upon in the course of neurosurgical operations, and which is probably duplicated in homologous areas of the two hemispheres, seems to have for its function the reproduction of (1) a remembered event or (2) thinking related to that event, and (3) the emotion it evoked’ (Horowitz, 1997).

On 1 September 1953, Dr. William Beecher Scoville performed bilateral mesial temporal lobe resections on a patient known as H.M. in the medical records. The inadvertent severe damage to the important limbic structures resulted in permanent loss of memory in this patient (Scoville, 1957). H. M. knew his name. He knew that his father’s family came from Thibodaux, LA, and his mother was from Ireland, and he knew about the 1929 stock market crash and World War II and life in the 1940s. But, he could remember almost nothing after that. Dr. Brenda Milner, professor of cognitive neuroscience at the Montreal Neurological Institute and McGill University studied H. M. almost up to his death in 2008 and noted: ‘He was a very gracious man, very patient, always willing to try these tasks I would give him and yet every time I walked in the room, it was like we’d never met’ (Carey, 2008).

Damage to discrete areas within the brain can thus produce a variety of disorders of the mind. ‘Taken together, the data from neurology suggests that despite our brain’s ability to organize our experience of ourselves and the world into a seamless unity, we are, in fact, made up of several parts, the loss of any of which can have dramatic effects on the whole’ (Craig, 2005).

In his Nobel Lecture, Sperry described the implications on concepts of the mind of the observations made after splitting the corpus callosum (Sperry, 1981). Sperry’s experiments, some conducted with R. E. Myers, showed that the cat with divided corpus callosum now had two *minds* either of which was capable of learning on its own, and of responding intelligently to changes in the world around it on its own. Subsequent experiments with rats, monkeys and later with human epileptic patients gave similar results. ‘Using *John Doe* as an example study, doctors examined *John Doe Left* and *John Doe Right*. Psychological tests showed that both John Does had remarkably similar personalities. Except for language ability, they were about as much alike as identical twins. Their attitudes and opinions seemed to be the same; their perceptions of the world were the same; and they woke up and went to sleep at almost the same times. There were differences however. *John Doe Left* could express himself in language and was somewhat more logical and better at [planning…]. *John Doe Right* tended to be somewhat more aggressive, impulsive, emotional - and frequently expressed frustration with what was going on.’ (McConnell, 1982). Such experiments led Sperry, Ornstein and others to conclude that each of the separated hemispheres has its own private sensations, perceptions, thoughts, feelings and memories, in short, that they constitute two separate minds, two separate spheres of consciousness (Gross, 2005). ‘Splitting the brain amounts to nothing less than splitting the self’ (Craig, 2005).

In addition to structure, we must consider the chemical processes within the brain. The effects of caffeine, alcohol, marihuana and opium on the brain and mind are common knowledge. Chemicals within the nervous system, such as adrenaline, serotonin, dopamine, the endorphins and encephalins, enable and modify the many functions of brain and mind and body we take for granted. Craig (2005) quotes the statement made by Steven Johnson: ‘Our personalities, the entities that make us both unique and predictable as individuals, emerge out of these patterns of chemical release.’

**Figure 1:**
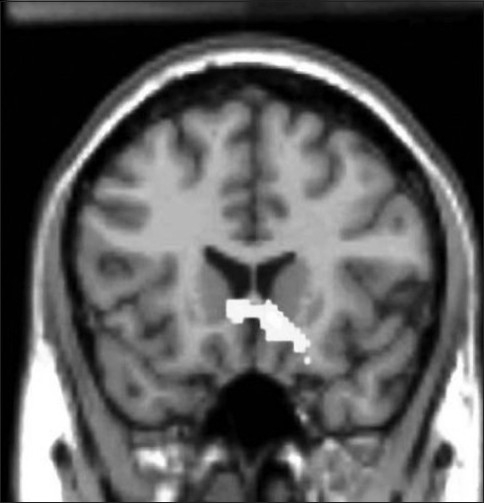
‘Nucleus accumbens activity in response to grief-related vs neutral words that was significantly greater in the complicated grief group compared to the non-complicated grief group’ (O’Connor *et al*., 2008).

Carter (1998) described modern techniques for mapping the brain and mind. ‘It is now possible to locate and observe the mechanics of rage, violence and misperception and even to detect the physical signs of complex qualities of the mind like kindness, humour, heartlessness, gregariousness, altruism, mother-love and self-awareness.’ O’Connor *et al*. (2008) studied the nucleus accumbens, the region most commonly associated with social attachment, in persons grieving from the death of a loved one [Figure 1].

Sounding a cautious note, Carter (1998) pointed out that whilst the optimist might wish for a complete understanding of human nature and experience from such studies, others may insist that a map of the brain can tell us no more about the mind than a terrestrial globe speak of Heaven and Hell.

To sum up, whilst the brain is ‘a physical mechanism, an arrangement of matter that converts inputs to outputs in particular ways’ (Pinker, 2003) the manner in which its hundred billion neurones are deployed, the infinite variations in their connections that result in very complex neural networks, the multitude of chemical and electrical reactions within it and the consequent almost unimaginable complexity of structure and function enable it to contain the mind just as it does the sources of all the other activities attributed to sentient life.

## Where is the Mind Located?

The brain is the organ of the mind just as the lungs are the organs for respiration.

## How does the Mind Function?

Krishnamoorthy (2009) uses an analogy based on computers to explain the workings of the mind: ‘The mind… is a virtual entity, one that reflects the workings of the neural networks, chemical and hormonal systems in our brain.’ The mind cannot be localised to particular areas within the brain, though the entire cerebral cortex and deep grey matter form important components. Consciousness, perception, behaviour, intelligence, language, motivation, drive, the urge to excel and reasoning of the most complex kind are the product of the extensive and complex linkages between the different parts of the brain. Likewise, abnormalities attributed to the mind, such as the spectrum of disorders dealt with by psychiatrists and psychologists, are consequences of widespread abnormalities, often in the chemical processes within different parts of the brain.

Two great British masters of neurology summed it up best.

John Hughlings Jackson (4 March, 1835–7 October, 1911) addressed anatomy.

‘Self, however, is dependent on the evolution of anatomically new structures. Jackson suggested that the evolutionary development of the prefrontal cortex is necessary to the emergence of self. In this sense it could be called the organ of mind. However, this is not to say that self resides in the prefrontal cortex. Rather, the new structure allows a more complex coordination of what is anatomically a sensori-motor machine.

‘In summary, Jackson conceived of the central nervous system as having a hierarchical organization that reflects evolutionary history. He used the terms lowest, middle, and highest centres…as proper names…to indicate evolutionary levels. Ascending levels show increasing integration and coordination of sensorimotor representations. The highest-level coordination, which allows the greatest voluntary control, depends on prefrontal activity. Self is a manifestation of this highest level of consciousness, which involves doubling. This doubling is established by the reflective capacity that enables one to become aware of individual experience in a way that gives a sense of an inner life.’ (Meares, 1999).

Sherrington (1961) addressed function and emphasised the limitations of our means for analysis:

‘Integration has been traced at work in two great, and in some respects counterpart, systems of the organism. The physico-chemical produced a unified machine… the psychical, creates from psychical data a percipient, thinking and endeavouring mental individual… they are largely complemental and life brings them co-operatively together at innumerable points… The formal dichotomy of the individual … which our description practiced for the sake of analysis, results in artifacts such as are not in nature… the two schematic members of the puppet pair… require to be integrated… This integration can be thought of as the last and final integration.’

## The Soul

### Introduction

The Bhagavad-Gita describes some of the qualities of the soul:
I say to thee weapons reach not the Life;
Flame burns it not, waters cannot o’erwhelm,
Nor dry winds wither it. Impenetrable,
Unentered, unassailed, unharmed, untouched,
Immortal, all-arriving, stable, sure,
Invisible, ineffable, by word
And thought uncompassed, ever all itself,
Thus is the Soul declared!
(Arnold, 1900)

***

Socrates – Now do you think one can acquire any particular knowledge of the nature of the soul without knowing the nature of the whole man?

Phaedrus – If Hippocrates the Asclepiad is to be trusted, one cannot know the nature of the body, either, except in that way. (Plato’s Phaedrus quoted by Prioreschi, 1996).

***

I wrote an essay called The Exact Location of the Soul (Selzer, 1976). I was being mischievous. I asked, ‘Is it under the kneecap or in a fold of the baby’s neck? Where is it?’ (Selzer Interview, 2005).

***

The search for the location of the human soul probably dates back to the awareness of such an entity. Termed *atman* by ancient Indian philosophers, *psyche* by the Greek and *anima* by the Romans, it has been considered resident within, but distinct from the human body. Many consider it immortal, postulating death to be the consequence of the departure of the soul from the body.

We use the term soul to denote essence as in the phrase ‘prayer is the very soul of religion.’ It is not surprising that we continue to enquire into the essence of man.

Several questions arise when considering the soul. Here are some examples. When does the soul enter the human body, as the sperm enters the egg or as they fuse into one cell or at a later stage? Does the soul influence the body, mind and intellect? Is the soul identical with what we term conscience? Since it animates the live person, does it govern functions of the body beyond the control of the mind, functions termed ‘vital’ by biologists? What happens to the soul during dreams, anaesthesia, trance-like states? What happens to it after the soul leaves the body? Where and how are acquired characters stored in the nebulous soul? Where, in the body, does the soul reside?

### Is there any point in searching for the location of the soul?

The answer must be in a resounding affirmative. The efforts over millennia to determine the nature and discover the location of the soul have resulted in a better understanding of the wonderful structure and function of man and his place in the cosmos.

In making this search and noting our findings, we must never lose sight of the cautionary note sounded by Leonardo da Vinci circa in 1487: ‘With what words O writer can you with a like perfection describe the whole arrangement of that of which the design is here?’ (MacCurdy 1956).

### The search and some conclusions

The physician-turned-author, Anton Chekhov (29 January, 1860–15 July, 1904) wrote to his friend Suvorin (7 May, 1889): ‘I think that when dissecting a corpse, the most inveterate spiritualist will be bound to ask himself, *Where is the soul here?* And if one knows how great is the likeness between bodily and mental diseases, and that both are treated by the same remedies, one cannot help refusing to separate the soul from the body.’ (See http://ebooks.adelaide.edu.au/c/chekhov/anton/c51lt/chapter24.html accessed on 6 December, 2010). Chekhov echoes the question asked by so many over the centuries.

Hippocrates concluded that madness originated in the brain. Plato (in *Timaeus*) felt that folly was a disease of the soul. Philistion subclassified folly into madness and ignorance (Harris, 1973).

Pythagoras (c. 570–c. 495 BC) had described the soul as consisting of three parts–intelligence, reason and passion. The seat of the soul extended from the heart to the brain, passion being located in the heart and reason and intelligence in the brain (Prioreschi, 1996).

**Figure 2:**
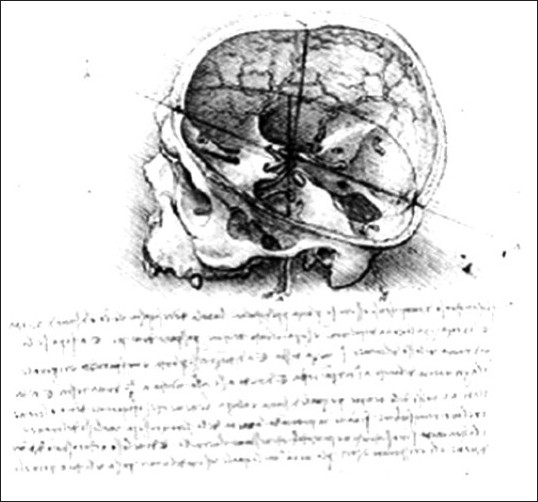
Leonardo depicted the location of the soul at the point where a series of intersecting lines meet (Santoro, 2009).

Leonardo da Vinci (1452–1519; see Figure 2), with his uncanny genius, placed the soul above the optic chiasm in the region of the anterior-inferior third ventricle (Santoro *et al*., 2009).

His summing up will continue to evoke admiration:

Though human ingenuity by various inventions with different instruments yields the same end, it will never devise an invention either more beautiful… than does Nature because in her inventions nothing is lacking and nothing superfluous and she… puts there the soul, the composer of the body, that is the soul of the mother which first composes in the womb the shape of man and in due time awakens the soul which is to be its inhabitant (Del Maestro, 1998).

**Figure 3:**
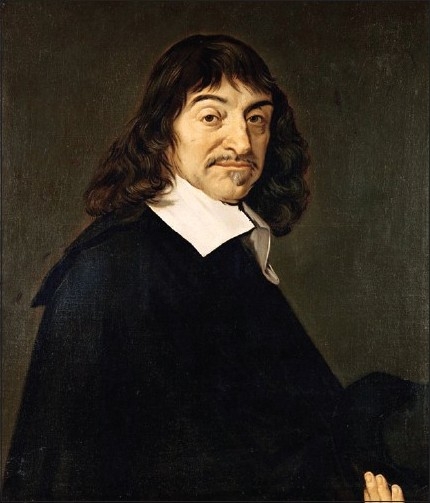
Portrait of Rene Descartes by Frans Hals, 1649.

René Descartes (1596–1650; see Figure 3) distinguished between the body and the soul, but equated the mind and soul:

There is a great difference between mind and body, inasmuch as body is by nature always divisible, and the mind is entirely indivisible. …When I consider the mind, that is to say, myself inasmuch as I am only a thinking being, I cannot distinguish in myself any parts, but apprehend myself to be clearly one and entire; and though the whole mind seems to be united to the whole body, yet if a foot, or an arm, or some other part, is separated from the body, I am aware that nothing has been taken from my mind. And the faculties of willing, feeling, conceiving, etc. cannot be properly speaking said to be its parts, for it is one and the same mind which employs itself in willing and in feeling and understanding. But it is quite otherwise with corporeal or extended objects, for there is not one of them imaginable by me which my mind cannot easily divide into parts. …This would be sufficient to teach me that the mind or soul of man is entirely different from the body, if I had not already been apprised of it on other grounds.

**Figure 4:**
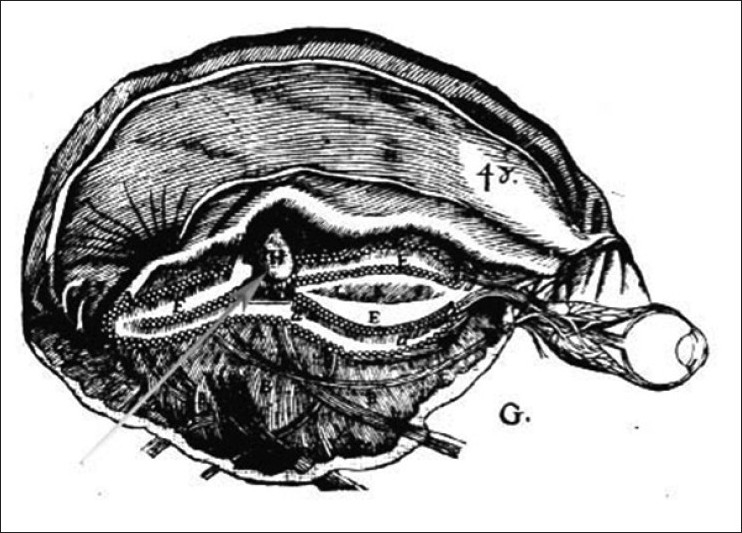
The pineal gland according to Descartes. This image from the 1664 edition of the Treatise of man illustrates Descartes’ view that the pineal gland (H) is suspended in the middle of the ventricles (Descartes 1664, p 63). (See http://plato.stanford.edu/entries/pineal-gland).

Descartes localised the soul in the pineal gland as it lay deep within the brain, in the midline and was unpaired [see Figure 4]. It is of interest that in neurosurgery journals, Descartes’ views are quoted with respect during discussions on surgery on the region of the pineal gland (Apuzzo, 1996).

Lancisi (1654–1720) agreed that the soul must lie deep within the brain, in the midline and in an unpaired structure, but favoured the corpus callosum, especially the *Nervali longitudinales ab anterioribus ad posteriora excurrentes*, which are still called the medial longitudinal striae of corpus callosum, or nerves of Lancisi. He felt that the vital spirits could flow in the fibres of the medial striae. These formed a pathway for the stream of the soul (or perhaps consciousness) between the anterior part of the corpus callosum and the anterior columns of the fornix and the posterior part of the corpus callosum and the thalami, a sort of connection between the seat of the soul and the peripheral organs, between the soul and the body (Di Ieva, 2007).

Thomas Willis (1621-1675) wrote *Cerebri Anatome* while being a Professor of Natural Philosophy in Oxford, where he used the anatomy of the brain as a tool to investigate the nature of the soul. In his dedication to *Cerebri Anatome*, he stated that the study of anatomy could ‘unlock the secret places of Man’s Mind and [to] look into the living and breathing Chapel of the Deity’ (O’Connor, 2003). He conceived of ‘a middle part of the brain, a kind of interior chamber of the soul… in the innermost part of which images or representations of all sensible things, sent in through the passages of the nerves… are revealed upon the corpus callosum… and so induce perception…’ Willis had considered as active powers of the soul ‘local motion, memory, phantasy and appetite’ which succeeded to ‘the passions’ (Clarke and O’Malley, 1996).

Albrecht von Haller (1708–1777) placed the soul in the medulla oblongata (Trimble, 2007; p27).

Bloom (2004) commented on the refutation of the dualist view differentiating the body and the soul:

… People often appeal to science to answer the question When does life begin? in the hopes that an objective answer will settle the abortion debate once and for all. But the question is not really about life in any biological sense. It is instead asking about the magical moment at which a cluster of cells becomes more than a mere physical thing. It is a question about the soul… It is not a question that scientists could ever answer. The qualities of mental life that we associate with souls are purely corporeal; they emerge from biochemical processes in the brain…

Santoro *et al*. (2009) recently reviewed the postulates regarding the nature and location of the soul in the human body. They concluded that there exist two dominant and, in many respects, incompatible concepts of the soul: one that understands the soul to be spiritual and immortal, and another that understands the soul to be material and mortal. In both cases, the soul has been described as being located in a specific organ or anatomic structure or as pervading the entire body, and, in some instances, beyond mankind and even beyond the cosmos.

Rationalists are doubtful. On the death of Harvard’s distinguished psychologist, Professor William James (1842–1910), Thomas Alva Edison (1847–1931) was asked about the human soul. ‘Soul? Soul? What do you mean by soul? The brain?’ ‘Well, for the sake of argument, call it the brain or what is in the brain. Is there not something immortal of or in the human brain – the human mind?’ asked Marshall. ‘Absolutely no.’ said Edison with emphasis:

‘There is no more reason to believe that any human brain will be immortal than there is to think that one of my phonographic cylinders will be immortal… No one thinks of claiming immortality for the cylinders… Then why claim it for the brain mechanism or the power that drives it? Because we do not know what that power is, shall we call it immortal? As well call electricity immortal because we do not know what it is… After death the force or power undoubtedly endures, but it endures in this world, not in the next. And so with the thing we call life, or the soul – mere speculative terms for a material thing which under given conditions drives this way or that. It too endures in this world, not the other. Because we are as yet unable to understand it, we call it immortal. It is the ignorant, lazy man’s refuge’ (Marshall, 1910).

What were William James’ views? He titled Lecture III of the published version of his Gifford Lectures *‘The reality of the unseen’* and discussed beliefs in objects that we cannot see. He quoted Immanuel Kant’s doctrine about such objects of belief as God and the soul as ‘properly not objects of knowledge at all.’ James referred to the strange phenomenon of a mind believing with all its strength in the real presence of a set of things of no one of which it can form any notion whatsoever (James, 1902).

In 1907, Dr. Duncan MacDougall of Haverhill, Massachusetts, decided to weigh the soul by weighing a human being in the act of death.

‘My first subject was a man dying of tuberculosis. It seemed to me best to select a patient dying with a disease that produces great exhaustion, the death occurring with little or no muscular movement, because in such a case the beam could be kept more perfectly at balance and any loss occurring readily noted.’ ‘The patient was under observation for three hours and forty minutes before death, lying on a bed arranged on a light framework built upon very delicately balanced platform beam scales. The patient’s comfort was looked after in every way, although he was practically moribund when placed upon the bed. He lost weight slowly at the rate of one ounce per hour due to evaporation of moisture in respiration and evaporation of sweat. During all three hours and forty minutes I kept the beam end slightly above balance near the upper limiting bar in order to make the test more decisive if it should come. At the end of three hours and forty minutes he expired and suddenly coincident with death the beam end dropped with an audible stroke hitting against the lower limiting bar and remaining there with no rebound. The loss was ascertained to be three-fourths of an ounce.’ He found the soul in six patients to weigh between 0.5 to 1.5 ounces (MacDougall, 1907).

In 1910, Dr. Max Baff of Clark University, Worcester, USA narrated to the correspondent of *The New York Times* his views on the use of x-ray cinematography to study the soul.

‘Even the activities of the so-called soul may be projected on the screen… Photographs might be taken at the moment of death and immediately after. It is the belief that when the heart stops beating the soul leaves the body. Something may be learned of the soul by observing the changes in its habitat, the marrow-like brain, at the moment when life ceases. I myself do not believe the soul to be a thing without the brain though I am neither an atheist nor an agnostic. However much people may believe that the soul is a separate thing, it must be borne in mind that its activities, thought and action, are confined within the limitations of the brain’ (Baff, 1910).

I am not aware of any success from Dr. Baff’s endeavours.

Otto Rank (2002) has summed the situation regards the soul well. He felt that belief in the soul grew out of the need to reassure ourselves of immortality, despite our knowledge of the immutable biological fact of death:

‘The collision (between our need and the fact of death) created a spark in our individual and social consciousness that through history has become both consolation and inspiration: the immortal soul… The immortal soul, whether fact or fiction, gives comfort.’

V. S. Ramachandran, brain scientist at the University of California, San Diego, is less tactful. He said in an interview that there might be soul in the sense of ‘the universal spirit of the cosmos,’ but the soul as it is usually spoken of, ‘an immaterial spirit that occupies individual brains and that only evolved in humans—all that is complete nonsense.’ Belief in that kind of soul ‘is basically superstition,’ he said (Dean, 2007).

For scientists who are people of faith, like Kenneth R. Miller, a biologist at Brown University, asking about the science of the soul is pointless, in a way, because it is not a subject science can address. ‘It is not physical and investigateable in the world of science,’ he said. Dr. Miller said he spoke often at college campuses and elsewhere and was regularly asked, ‘What do you say as a scientist about the soul?’ His answer, he said, is always the same: ‘As a scientist, I have nothing to say about the soul. It’s not a scientific idea’ (Dean, 2007).

### If there be a soul, where is it located? Views of neuroscientists

If we accept the existence of the soul and its localisation in the brain, we must focus on the brainstem. Christopher Pallis (1983), discussing the definition of whole-brain death, provided a modern concept of the soul. ‘The loss of the capacity for consciousness and of the capacity to breathe (after brain death) relate to functional disturbances at the opposite ends of the brain stem while the former is also a meaningful alternative to “the departure of the soul”.’

Greenfield’s (1997) description is relevant. The soul, like the seat of consciousness (in its neurological sense) lies in ‘the cocktail of brain soup and spark’ within the deep cerebrum and brainstem, whence dopamine, noradrenaline, acetylcholine are released ‘in a fountain-like arrangement on to the more sophisticated regions of the (cerebral) cortex and immediate subcortical structures’ to produce a series of electrical and chemical events.

Neurosurgeons operating within the brainstem are known to tell their postgraduate students: ‘I need not emphasise the need for the greatest accuracy and delicacy when operating here – we are now in the abode of the soul.’ (This is the gist of what I have heard when watching some very senior neurosurgeons perform delicate operations deep within the brain.)

We must confess that the existence of the soul remains unproven by tests ‘in the acid baths of experiment and logic.’ Nor has it ‘enjoyed repeated vindication’ (Wilson, 1998). Despite all that has been written on the soul, it is difficult to fault Musil’s observation published in 1990: ‘(There is) an abiding miscommunication between the intellect and the soul. We do not have too much intellect and too little soul, but too little intellect in matters of soul.’

Perhaps, we shall eventually come to conclusions similar to those reached by Sir Thomas Browne (19 October, 1605–19 October, 1682) in his most famous work, the *Religio Medici*:

‘Amongst all those rare discoveries and curious pieces I find in the Fabrick of Man, there is no Organ or Instrument for the rational Soul; for in the brain there is not anything of moment more than I can discover in the crany of a beast, and this is an argument of the inorganity of the Soul. Thus we are men, and we know not how; there is something in us that can be without us, and will be after us; though it is strange that it hath no history what it was before us, nor cannot tell how it entered in us’ (Browne, 1635/2009).

We remain ‘children of Tantalus, frustrated by the failure to grasp that which seems within reach…’ (Wilson, 1998).

Of course, if you have a hyperactive funny bone, you could paraphrase Woody Allen, who, as so often, has the ultimate comic word on the subject: ‘You cannot prove the non-existence of the soul; you just have to take it on faith.’ (http://cavett.blogs.nytimes.com/2007/02/07/ghost-stories/?apage=3)

## Concluding Remarks [see also Figure 5]

**Figure 5:**
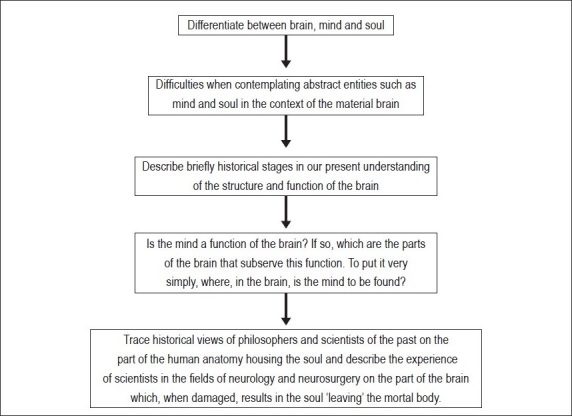
Flowchart of paper

The mind and the soul remain fascinating enigmas. Whilst we have made some progress in our understanding of these two hazy constituents of life, much is as yet poorly understood.

Religious scholars ask us scientists to desist from any attempt at studying the soul. Hindu philosophers tell us that the soul of a person who has attained *moksha* (liberation from the cycle of re-birth) unites with God. The soul has often been termed the God within each of us.

The spirit of enquiry that is the essence of science must stimulate us to continue our efforts at understanding it better. If, in doing so, we understand God better, this can only be to our advantage.

### Take home message

The study of the brain, mind and soul has engaged some of the finest intellects of yesteryears. It remains an ennobling and inspiring pursuit, worthy of all those who are dedicated votaries of science.

## Questions That This Paper Raises

1. What are the precise definitions of mind and soul?
2. Do you agree with the author’s conclusions on the mind in the brain?
3. Which of the many modern tools used in the study of the brain should we use to further our understanding of the mind?
4. Most religious texts treat the soul as ‘something’ that leaves the human body at death. What is this ‘something’ and if it leaves the human body, where is it located during life?
5. Philosophers have argued that the soul is not amenable to scientific scrutiny. Accepting this point of view, would mean an end to any serious exploration of this hitherto nebulous entity. What studies can we undertake to advance our knowledge and understanding?

### About the Author

Sunil Pandya is a neurosurgeon and thinker on medical ethics. He joined the Grant Medical College in 1957 and trained at the Sir JJ Group of Hospitals, Mumbai. He obtained the MBBS (1961), MS (1965) and Fellowship of the National Academy of Medical Sciences. He underwent further training under Professor Valentine Logue at the Institute of Neurology, London. He joined Dr Homi Dastur at the Department of Neurosurgery, Seth G.S. Medical College and KEM Hospital in 1967 as a Pool Officer and was appointed to the staff as Asst Neurosurgeon in 1968. In 1975, on Prof Dastur’s retirement, he was appointed Prof of Neurosurgery. He retired on superannuating in 1998, and has since worked at the Jaslok Hospital and Research Centre, Mumbai. He is Editor Emeritus, Indian Journal of Medical Ethics; Journal Ombudsman, Journal of Post Graduate Medicine; on the Editorial Board, National Medical Journal of India; and on the International Editorial Advisory Board of the Mens Monographs.
